# Artificial Intelligence-Based Modeling to Aid in the Identification of Genetic Conditions in Infants with Congenital Heart Disease

**DOI:** 10.64898/2026.10.12.26365328

**Published:** 2026-10-13

**Authors:** Michael Caiola, Tyler Thompson, Sara B. Stephens, Bita Salamat, Krista Harris, Claudia Lara, Zeynep Coban Akdemir, Lilei Zhang, Matthew Roth, Aleksandar Milosavljevic, Andrew C. Weitz, Shaine A. Morris

## Abstract

**Background:**

Genetic conditions are common in congenital heart disease (CHD) and affect care, but are often missed or diagnosed late. We asked whether routine clinical data can shortlist the most likely genetic conditions.

**Methods:**

Retrospective single-center cohort of children with CHD and an echocardiogram who had either a confirmed genetic diagnosis or negative cytogenetic testing and CHD gene sequencing. Echocardiogram images and serial medical record data were added as model inputs in the order they become clinically available. Class-balanced logistic regression (LR) and a multimodal deep learning fusion network were fitted to the same inputs at each step. The primary metric was balanced top-3 accuracy (correct diagnosis among the top three, averaged across conditions), with bootstrap intervals.

**Results:**

The cohort included 1,720 children (988 with common CHD-associated genetic conditions, 231 rare diagnoses, 501 negative testing). Using only inputs generated at the echocardiogram visit, LR reached a balanced top-3 accuracy 0.758 (chance 0.429). Serial records, radiology notes and ICD-10 codes, raised this to 0.809. Multimodal fusion performed similarly at each stage. With a genetic condition withheld from training, the fusion embedding placed affected children nearest to others sharing a gene group in 0.37 of cases versus 0.10 without the embedding (+0.268 [95% CI +0.242, +0.295]).

**Conclusions:**

Routine clinical data available at first echocardiogram can help identify likely genetic diagnoses, and later coded inputs ceiling. Multimodal fusion added no accuracy over LR, but places children sharing a genetic cause near one another, making nearest-neighbor retrieval informative for undiagnosed children.

